# Reconstruction of elasticity: a stochastic model-based approach in ultrasound elastography

**DOI:** 10.1186/1475-925X-12-79

**Published:** 2013-08-10

**Authors:** Minhua Lu, Heye Zhang, Jun Wang, Jinwei Yuan, Zhenghui Hu, Huafeng Liu

**Affiliations:** 1 National-Reginoal Key Technology Engineering Laboratory for Medical Ultrasound, Guangdong Key Laboratory for Biomedical Measurements and Ultrasound Imaging, Department of Biomedical Engineering, School of Medicine, Shenzhen University, Shenzhen, China; 2 Key Lab for Health Informatics of the Chinese Academy of Sciences, Institute of Biomedical and Health Engineering Shenzhen Advanced Institutes of Technology, Chinese Academic of Sciences, Shenzhen, China; 3 State Key Laboratory of Modern Optical Instrumentation, Department of Optical Engineering, Zhejiang University, Hangzhou, China

## Abstract

**Background:**

The convectional strain-based algorithm has been widely utilized in clinical practice. It can only provide the information of relative information of tissue stiffness. However, the exact information of tissue stiffness should be valuable for clinical diagnosis and treatment.

**Methods:**

In this study we propose a reconstruction strategy to recover the mechanical properties of the tissue. After the discrepancies between the biomechanical model and data are modeled as the process noise, and the biomechanical model constraint is transformed into a state space representation the reconstruction of elasticity can be accomplished through one filtering identification process, which is to recursively estimate the material properties and kinematic functions from ultrasound data according to the minimum mean square error (MMSE) criteria. In the implementation of this model-based algorithm, the linear isotropic elasticity is adopted as the biomechanical constraint. The estimation of kinematic functions (i.e., the full displacement and velocity field), and the distribution of Young’s modulus are computed simultaneously through an extended Kalman filter (EKF).

**Results:**

In the following experiments the accuracy and robustness of this filtering framework is first evaluated on synthetic data in controlled conditions, and the performance of this framework is then evaluated in the real data collected from elastography phantom and patients using the ultrasound system. Quantitative analysis verifies that strain fields estimated by our filtering strategy are more closer to the ground truth. The distribution of Young’s modulus is also well estimated. Further, the effects of measurement noise and process noise have been investigated as well.

**Conclusions:**

The advantage of this model-based algorithm over the conventional strain-based algorithm is its potential of providing the distribution of elasticity under a proper biomechanical model constraint. We address the model-data discrepancy and measurement noise by introducing process noise and measurement noise in our framework, and then the absolute values of Young’s modulus are estimated through the EFK in the MMSE sense. However, the initial conditions, and the mesh strategy will affect the performance, i.e., the convergence rate, and computational cost, etc.

## Introduction

In the daily clinical exercises, palpation is frequently used as one part of physical examination to determine suspicious lesion’s size, shape, firmness, or location [1], but this process is largely decided by the doctor’s experiences. Therefore, ultrasound elastography [2], which aims to replace subjective palpation process, is developed to provide quantitative measures of material properties of tissue. As an evolving imaging modality, ultrasound elastography has already been broadly evaluated in the clinical practices and shown great potentials in providing valuable diagnostic information, such as improving the diagnostic accuracy of breast and prostate cancer [3-9], assessing plaque vulnerability [10-13] and guiding minimally invasive therapy [14-16]. Similar elastography techniques have been also developed using magnetic resonance imaging [17-19] and mammographic imaging [20]. Ultrasound elastography can be broadly grouped into quasi-static ultrasound elastography and dynamic ultrasound elastography according to the mechanical excitation applied on the biological tissue [21]. During the quasi-static ultrasound elastography, which is the focus of this paper, the mechanical excitation is usually applied through the ultrasound transducer and this external mechanical stimuli can be considered as a steady-state quasi-static loading [2]. After the deformation caused by the external loading is captured by the ultrasound machine, the elastographic image can be generated using different reconstruction techniques [22]. During the dynamic ultrasound elastography [3,23], harmonic excitations on the order of 10–1000 *Hz*, or transient dynamic excitations are applied to the biological tissue in order to generate the shear wave. After the shear wave of the biological tissue is captured by the ultrasound machine, the following images of shear modulus can be generated using similar reconstruction techniques [24].

In spite of varieties of ultrasound elastography, we focus on the estimation of mechanical elasticity in quasi-static ultrasound elastography, which is one popular elastographic technique because of its efficiency and conveniency. The imaging process of quasi-static ultrasound elastography can be described by following four steps: a radio-frequency (RF) echo frame is first acquired from the tissue; second, a small deformation is induced into the tissue through the probe; third, a second RF frame is acquired from the deformed tissue; fourth, the displacement field, strain field and the distribution of tissue elasticity are reconstructed. Numerous techniques have been proposed to solve the reconstruction problem in step four of quasi-static ultrasound elastography in the last decades [25]. According to the way to obtain the tissue elasticity, the existing reconstruction algorithms for step four in quasi-static ultrasound elastography can be classified into two groups: the direct approach and iterative approach [26,27]. However, the estimation of stable and meaningful mechanical elasticity in ultrasound elastography still remains as a challenging researching topic at present because of its ill-posed nature [28]. In the recent review [22], the model-based approach is considered as one promising researching direction in solving the reconstruction problem in step four, and how to define the meaningful model constraint during the quasi-static ultrasound elastography is the key issue in the model-based approach.

According to the constitute law of elasticity, accurate quantification of material properties, such as Young’s modulus, is feasible only when the distribution of all components of the internal stress is known. In quasi-static ultrasound elastography, however, the distribution of internal stress is hardly available. Hence, in the direct approach, the elastographic image is reconstructed always by converting the strain value as relative Young’s modulus using a very simple model constraint (e.g., Hook’s law) [2]. Mostly, the distribution of relative Young’s modulus (e.g., inverse strain values), is directly interpreted as the elastographic image based on the assumption of stress uniformity. But the assumption of uniformed internal stress distribution is not always valid in clinical cases. Furthermore, poor contrast-transfer efficiency will incur because of the assumption of uniformed stress distribution. In [2], an artifact, called “target hardening”, caused by this simplified assumption has been discussed. In the following work [29], an analytic solution of the elasticity equations derived for an infinite medium subjected to a uniaxial compression induced by a finite size compressor was used to verify the fact that a gradual decay of stress with increasing distance along the axis and off axis would cause “target hardening”. A different approach later tried to compress the target hardening by reconstructing the elastogram with measured strain and predicted stress field [30]. However, this approach still contained artifacts in an inhomogeneous medium: bi-directional shadows appear at the boundaries of the inclusion and the quality of elastographic image is even worse in complex situations. Several other groups including Doyley et al. [31], have sought for better quality of elastographic images by exploring the feasibility of solving the inverse problem based on a theoretical representation of forward elasticity model without requiring the internal stress distribution, but it turned out to be difficult in recovering the elastic modulus by inverting the forward elasticity model. In [32] and [33], a similar technique for quantifying the tissue elasticity was proposed to invert a finite difference representation of forward elasticity model with the pressure terms disappearing in the equations elimination, but artifacts were still observed in the reconstructed images. In another approach [34], the pressure term was included, but it was found that the method failed to employ in practice since hydrostatic pressure is nearly impossible to be measured from within tissues. In a word, the direct approach is trying to simplify the inverse problem (i.e., reconstruction of elastographic images), by inverting the forward model directly using strict controlled conditions or simplified assumptions, but these conditions or assumptions are usually invalid in clinical situation. For example, the assumption of internal stress uniformity within tissue is seldom valid in clinical cases. Overall, the direct approach’s capability is limited to provide the relative elastic modulus, but the ultimate aim of elastography to reconstruct the mechanical elasticity of tissues. What’s more, the differentiating process to compute the strain map from noisy displacement also negatively degrades the accuracy of direct approach because of its one-time-transformation process though the direct method still attracts great attentions from many groups at present because of its efficiency.

To overcome the drawbacks of direct methods, an alternative approach to replace the direct inversion technique is the iterative approach, which can be considered as a repeated direct inversion process, but with much more reasonable constraints, until an optimal estimation of the distribution of meaningful elastic modulus is obtained. Two key features of the iterative approach are: an iterative procedure and an external constraint. In [35], a Newton Raphson iterative scheme and a Tikhanov regularization method was applied to stabilize the reconstruction in the presence of noise. A similar algorithm was adopted in [36] and [26] for solving the inverse problem to recover the elastic modulus using Gauss-Newton algorithm, but this algorithm would cost heavy computation because of forming a Jacobian matrix. In order to reduce the computational cost from the iterative procedure, the gradient through adjoint elasticity equations was calculated and then the direction of gradient descent was used to find the optimal estimation [37]. In order to better recover smooth kinematic functions, one iterative two-dimensional phase-matching method was developed to reconstruct a two-dimensional displacement vector field during acquisition of two successive RF-echo data frames [38]. Another work employed a split-and-merge strategy, which used the strain images to provide a priori knowledge of relative stiffness distribution to reduce the computation time and avoid singularity in solving the inverse problem, to estimate the elastic modulus over the image sequence [39]. In the direct approach, simplified constitutive models can be directly inverted, but easily destroyed by the noises from measurement and complex tissue deformation. Therefore, the mechanical models with the ability of self-correcting to minimize the cost function that measures the goodness of fit between computed and measured data are always adopted in the iterative approach. For example, a finite element (FE) model with appropriate mathematical description of the object was adopted as the constraint to reconstruct the modulus of elasticity in [23]; in prostate elastography or intravascular elastography where the compression direction is radial, one FE-based approach was developed to study the reconstruction of tissue elasticity for ultrasound elastography in the polar coordinates [40]; since the material properties of lesion is important to the FE model, one iterative approach that used a FE model was developed to reconstruct the Young’s modulus from the measured force-displacement slope [41].

The iterative approach with a biomechanical model constraint was even named as a model-based method in [28]. In [28], a comparative evaluation of the model-based method and direct approach was conducted, and a conclusion that better contrast transfer efficiency could be achieved particularly for the high-contrast inclusions. However, the model-based method is fraught with three major defects: model-data discrepancy, measurement noise and no unique result. The model mismatch and the measurement noise will undoubtedly compromise the quality and accuracy of the ensuing elastograms. The reason for non-unique solution is that there is no guarantee of producing unique modulus elastograms as the ill-posed nature of inverse elasticity problems. Nevertheless, with the assistance of iterative procedure and meaningful biomechanical model, the iterative approach should be able to perform better than direct methods for the recovery of absolute values of elastic modulus if three major defects can be properly handled.

To overcome the defects of the mode-based approach mentioned above, we have introduced a stochastic model-based algorithm in our previous paper [42]. Different from earlier approaches, this algorithm treats the displacement and Young’s modulus as random variables that need to be estimated together from ultrasonic RF signals: an EKF runs iteratively until it converges to the steady state of deformed tissue, and the optimal estimation of the full displacement field and Young’s modulus are computed in a MMSE sense. Furthermore, the model-data discrepancy and measurement noise are treated as white noises after the biomechanical model is transformed into the stochastic state space. However, in [42], we only present qualitative comparison in the preliminary results. In this paper, three groups of data, synthetic data, phantom data and patient data, are used to show the ability of our framework in recovering the absolute values of elastic modulus. The results and quantitative comparisons prove that this algorithm is a practicable way to suppress artifact noise. In the end, one fact should be pointed out that it is straightforward to apply our strategy to other forms of quasi-static elastography, but only two dimensional quasi-static ultrasound elastography are studied in this paper because of limited space.

The rest of this paper is organized as follows: section ‘Methodology’ describes the details of our elasticity reconstruction method: the linear elasticity and its finite element implementation are introduced, followed by the discussion about the integration of the stochastic state space strategy and the dynamic equation for the quasi-static ultrasound elastography. In section ‘Experiments and results’, the results obtained from the experiments using simulated data, real phantom data and in-vivo clinical data are presented and discussed respectively. In each set of data, our results are compared to the strain-based maps. As the ground truths are available in simulation and phantom studies, the accuracy and robustness of our framework are evaluated by comparing the results generated by our strategy to the ground truths. In the clinical experiment, the elastographic images are compared to CT images with lesions indicated by the doctor. Finally, the discussion of our framework, concluding remarks and future research endeavors are outlined in section ‘Discussion’.

## Methodology

### Linear elasticity

In order to construct a meaningful, yet computationally feasible computing scheme, material properties of the tissue should be properly modeled. In general, the biological tissue is a non-rigid object that deforms over time and has very complicated material properties in terms of the underlying constitutive laws [43]. However, in this paper, we only study the quasi-static ultrasound elastography, which is to utilize a steady state quasi-static excitation on the tissues using the ultrasound transducer. Since the tissue is compressed slightly during quasi-static elastography, constitute law of linear elasticity is still valid [2]. Hence, for computational simplicity, in our current two dimensional implementation, the linear isotropic continuum material is adopted to describe the mechanical behavior of the tissue during the quasi-static elastography, and the relationship of the stress *σ* and the strain *ε* obeys the Hooke’s law [44]:

(1) $$\begin{array}{l} \sigma =S\varepsilon \end{array}$$

where *S* is the strain-stress matrix.

For the two dimensional case discussed in this paper, let *u*(*x*, *y*) and *v*(*x*, *y*) be the displacement along the *x*- and *y*-axis of a point, the infinitesimal strain tensor *ε* of the point is:

(2) $$\begin{array}{lcrr} \varepsilon =\left[\begin{array}{c} \frac{\mathrm{\partial u}}{\mathrm{\partial x}}\\ \frac{\mathrm{\partial v}}{\mathrm{\partial y}}\\ \frac{\mathrm{\partial u}}{\mathrm{\partial y}}+\frac{\mathrm{\partial v}}{\mathrm{\partial x}} \end{array}\right] & & & \end{array}$$

In two-dimensional situation, under the plane strain condition [44], strain-stress matrix *S* can be derived as:

(3) $$\begin{array}{lcrr} S=\frac{E}{\left(1+\nu )(1-2\nu \right)}\left[\begin{array}{ccc} 1-\nu & \nu & 0\\ \nu & 1-\nu & 0\\ 0 & 0 & \frac{1-2\nu }{2} \end{array}\right] & & & \end{array}$$

*S* is a material-dependent matrix, *E* stands for the Young’s modulus, and *ν* stands for the Poisson’s ratio. Here, the Young’s modulus *E* and the Poisson’s ratio *ν* are two material-specific parameters. This fact that the internal stress caused by the deformation is a function of the displacement vector and the material parameters is quite clear from these equations. Ideally, the problem could be tackled in three dimension to avoid the through-plane motion effect, but in this paper we only study the two-dimensional problem because the images of quasi-static ultrasound elastography are two dimensional data collected by the linear array of ultrasonic probe. Further, a linear material model is used to illustrate the basic ideas and rationales of a joint estimation strategy to recover the distribution of real elasticity for quasi-static ultrasound elastography, but more realistic models can be easily adopted into current framework in the future works.

### Stochastic finite element method

In the past decades, the deterministic finite element method has been able to provide an effective and convenient platform for biomechanical studies in the past decades [45,46]. However, it does not have the capability to process the uncertainties of material properties, and kinematic observations, i.e., measurements of the displacement. Especially the ultrasound imaging-derived data are usually corrupted by noises of various nature. It is thus necessary to develop a strategy, the stochastic finite element method (SFEM), which has been used widely for structural dynamic analysis in probability analysis frameworks [47,48], to process the data from the ultrasound elastography. The main difference between SFEM and finite element method (FEM) is that material properties are deterministic variables in FEM, but are described by random fields, possibly with known prior statistics in SFEM.

In order to build our implementation of SFEM for the quasi-static ultrasound elastography, a Delaunay triangulated finite element mesh is constructed at the first image frame before compression. An isoparametric formulation defined in a natural coordinate system is used, where, for tri-nodal linear element, the basis functions are linear functions of the nodal coordinates [44]. With mesh constructed, assuming that the material parameters *E* (Young’s modulus) and *ν* (Poisson’s ratio) are temporally constant but varying spatially, we can have the following dynamic governing equation [44]:

(4) $$\begin{array}{lcrr} MÜ+C\dot{U}+\text{KU}=R & & & \end{array}$$

where *M*, *C* and *K* are the mass, damping and system stiffness matrices, *R* is the load vector, and *U* is the displacement vector. In this work, *U* consists of *u*(*x*, *y*) and *v*(*x*, *y*). The calculation of *M*, *K* and *C* matrices are the standard FEM procedure, which can be read in [44]. Because the tissue density can be generally considered to be uniform over the region of interest, *M* is a known function of the material density and is temporally and spatially constant. *K* is a function of the constitutive law of material, which is linear elasticity here, and is related to the material-specific Young’s modulus and Poisson’s ratio, which are considered as constants temporarily in this study. In our framework, these two local material parameters are treated as random variables with known a priori statistics, and will be estimated along with the motion parameters. The damping matrix C is frequency dependent, and we assume small proportional Rayleigh damping with *C* = *αM* + *βK* in our implementation. In Rayleigh damping *α* is the mass proportional Rayleigh damping coefficient and *β* is the stiffness proportional Rayleigh damping coefficient [44]. In practice, it is difficult to determine the damping parameters because they are frequency dependent. We assume Raleigh damping for the very low damping biological tissue during quasi-static elastography, and fix the two weighting constants to be 1%.

### State space representation

In order to employ our filtering strategy to integrate biomechanical model, the dynamics equation (Equation (4)) needs to be transformed into a state-space representation of the continuous-time linear stochastic system. First the kinematic vector *x*(*k*) and the material parameter vector *θ* are defined as:

(5) $$\begin{array}{lcrr} x(t)=\left[\begin{array}{c} U(t)\\ \dot{U}(t)\; \end{array}\right] & & & \end{array}$$

(6) $$\begin{array}{lcrr} \;\theta =\;\left[\begin{array}{c} E\;\;\;\;\;\;\\ \nu \;\;\;\;\;\; \end{array}\;\right] & & & \end{array}$$

Where the kinematic vector *x*(*t*) is consisted of displacement *U*(*t*) and velocity $\dot{U}(t)$ information, and the material parameter vector *θ* is consisted of Young’s modulus *E* and Poisson’s ratio *ν*. In general, tumor inclusion or tissue blocked from its blood nutrients is stiffer than normal tissue, which mostly reflects in the variation of *E*. Since benign and cancerous tumors usually have distinguishing elastic properties (i.e., different Young’s modulus value), the value of Poisson’s ratio can be fixed in the following implementation. For example, as one of incompressible materials, the Poisson’s ratio of tissue can be set close to 0.5.

The state space representation of Equation (4) becomes

(7) $$\begin{array}{lcrr} \dot{x}(t)=A_{c}x(t)+B_{c}W(t)+n(t) & & & \end{array}$$

where *n*(*t*) is the process noise which is an additive, zero-mean, white noise (*E*[*n*(*t*)] = 0; *E*[*n*(*t*)*n*(*s*)^′^] = *Q*_*n*_(*t*)*δ*_*ts*_, where *Q*_*n*_ is the process noise covariance). Process noise cannot be ignored if model-data discrepancy exists. The system matrices *A*_*c*_, *B*_*c*_ and input forces *W*(*t*) are:

() $$\begin{array}{llll} A_{c} & = & \left[\begin{array}{cc} 0 & I\\ -M^{-1}K & -M^{-1}C \end{array}\right] & \\ B_{c} & = & \left[\begin{array}{cc} 0 & 0\\ 0 & -M^{-1} \end{array}\right] & \\ W(t) & = & \left[\begin{array}{c} 0\\ R(t) \end{array}\right] & \end{array}$$

An associated measurement equation, which describes the observations *y*(*t*) extracted from the ultrasonic RF data, can be expressed in the form:

(8) $$\begin{array}{lcrr} y(t)=\text{Hx}(t)+e(t) & & & \end{array}$$

where *e*(*t*) is the measurement noise which is additive, zero mean, and white (*E*[*e*(*t*)] = 0;*E*[*e*(*t*)*e*(*s*)^′^] = *R*_*e*_(*t*)*δ*_*ts*_, where *R*_*e*_ is the measurement noise covariance), independent of *n*(*t*). Although the noise in the displacement measurement extracted from ultrasonic RF data might not be white Gaussian noise, the property of the noise from ultrasonic RF data is rarely available due to the complicated collecting process of ultrasonic RF data. Hence our best guess is to assume it is white Gaussian noise, which is one common treatment in engineering field [49-51]. Furthermore, we found that the performance of this assumption is satisfied in the following experiments. *H* is the measurement matrix which should be specified by the relation between state vector *x*(*t*) and measurement vector *y*(*t*).

In order to run the dynamic equation (Equation (4)) in computer, it should be transformed into a discrete state-space equation, typically seen in control and estimation literature [50,52]. We discretize Equation (7) and (8) over a constant time interval *T*. Since the interval *T* is always a known constant, we can replace *kT* with *k* in following equations

(9) $$\begin{array}{lcrr} x(k+1)=\text{Ax}(k)+\text{Bw}(k)+n(k), & & & \end{array}$$

where

(10) $$\begin{array}{lcrr} A & = & e^{A_{c}T}, & \end{array}$$

(11) $$\begin{array}{lcrr} B & = & A_{c}^{-1}(e^{A_{c}T}-I)B_{c} & \end{array}$$

The associated discrete measurement equation is:

(12) $$\begin{array}{lcrr} y(k)=\text{Dx}(k)+e(k) & & & \end{array}$$

where *y*(*k*) is the measurement vector contained the displacement extracted from ultrasound RF data. In most quasi-static elastography, only the axial components of displacement vector are extracted. Therefore, the corresponding place of *D* will be set to 1 or 0 according to the available measurement data. *n*(*k*) and *e*(*k*) are process noise and the measurement noise respectively.

In order to perform the estimation of the material parameter distribution, the state vector *x* is augmented by the material parameter vector *θ* to form the new state vector *z*:

(13) $$\begin{array}{lcrr} z={\left[x,\theta \right]}^{T} & & & \end{array}$$

Accordingly, the new augmented state equation is:

(14) $$\begin{array}{lcrr} z(k+1)=f(z(k),w(k))+v_{s}(k) & & & \end{array}$$

with

(15) $$\begin{array}{lcrr} f(z(k),w(k))=\left[\begin{array}{c} A(\theta )x(k)+B(\theta )w(k))\\ \theta \end{array}\right] & & & \end{array}$$

(16) $$\begin{array}{lcrr} v_{s}(k)=\left[\begin{array}{c} v(k)\\ 0 \end{array}\right] & & & \end{array}$$

Where *v*(*k*) is the process noise, introduced by the model mismatch. The new augmented measurement equation is derived:

(17) $$\begin{array}{lcrr} y(k)=h(z(k))+e_{o}(k), & & & \end{array}$$

with

(18) $$\begin{array}{lcrr} h(z(k))=[D,0]\left[\begin{array}{c} x(k)\\ \theta (k) \end{array}\right] & & & \end{array}$$

(19) $$\begin{array}{lcrr} e_{o}(k)=e(k) & & & \end{array}$$

Using previous assumptions of noises, the augmented noises are also Gaussian with distribution:

(20) $$\begin{array}{lcrr} v_{s}(k)∼N(0,Q_{s}),\;\text{where}\;Q_{s}=\left[\begin{array}{cc} Q_{v} & 0\\ 0 & 0 \end{array}\right] & & & \end{array}$$

(21) $$\begin{array}{lcrr} e_{o}(k)∼N(0,R_{o}),\;\text{where}\;R_{o}=R_{e} & & & \end{array}$$

### Extended kalman filter (EKF) for joint state estimation

The reconstruction of quasi-static elastographic image now can be defined as calculating the optimal estimates of the nonlinear state-space estimation problem represented by the Equation (14) and (17) above. This leads to solve this nonlinear estimation problem using the EKF framework, which is to linearize the augmented state (Equation (14)) at each time step and perform a recursive procedure with natural block structure to estimate the material parameter. EKF operates like Kalman filter, adopting the same form of feedback control in estimation: the filter makes an estimation of the state at one moment and then obtains feedback in form of measurements. The operations of EKF can be divided into two steps: time update equations and measurement update equations. Time update equations are responsible for projecting forward the current state and error covariance estimates to obtain the priori estimates for the next time step, while the measurement update equations are responsible for the feedback with the posterior data.

Initializing the filter with $\hat{z}{\;}^{-}(0)={\hat{z}}_{0}$ and $P(0)=\underset{0}{\sum }$, the EKF runs sequentially as follows: 

(1) compute the prior estimation of the state, from *t*−1 to *t*:

(22) $$\begin{array}{ll} \hat{z}{\;}^{-}(t)=f(\hat{z}(t-1),w(t)), & \; \end{array}$$

(2) project the error covariance from *t*−1 to *t*:

(23) $$\begin{array}{ll} P{\;}^{-}(t)=F_{t}P(t-1)F_{t}^{T}, & \; \end{array}$$

(3) compute the Kalman gain at *t*:

(24) $$\begin{array}{ll} G(t)=P^{-}(t)H^{T}{\left(HP^{-}(t)H^{T}+R_{0}\right)}^{-}, & \; \end{array}$$

(4) compute posterior estimation of the state with the measurement at *t*:

(25) $$\begin{array}{ll} \hat{z}(t)=\hat{z}{\;}^{-}(t)+G(z(t)-h(\hat{z}{\;}^{-}(t))), & \; \end{array}$$

(5) update the error covariance at *t*:

(26) $$\begin{array}{ll} P(t)=(I-G(t))(z(t)-h(\hat{z}{\;}^{-}(t))), & \; \end{array}$$

(6) repeat (1)-(5) to convergence. The needed quantities for Equations (22)–(26) are defined by:

(27) $$\begin{array}{ll} F_{t}=\frac{\partial }{\partial }f(z(t),w(t)){|}_{z=\hat{z}}=\left[\begin{array}{cc} A(\hat{\theta }) & M_{t}\\ 0 & I \end{array}\right] & \; \end{array}$$

(28) $$\begin{array}{ll} H(\;\hat{z}(t))=\frac{\partial }{\partial }h(z(t)){|}_{z=\hat{z}}=\left[\begin{array}{cc} D & 0 \end{array}\right] & \; \end{array}$$

(29) $$\begin{array}{ll} M_{t}=\frac{\partial }{\partial \theta }(A(\theta )\hat{x}(t)+B(\theta )w(t)){|}_{\theta =\hat{\theta }} & \; \end{array}$$

In our implementation, the initial error covariance matrix *P*(0) consists of four parts:

(30) $$\begin{array}{ll} P(0)=\left[\begin{array}{cc} P_{1}(0) & P_{2}(0)\\ P_{2}^{T}(0) & P_{3}(0) \end{array}\right] & \; \end{array}$$

*P*_1_(0), *P*_3_(0) are the sub-matrices inside the initial error covariance matrix related to the trustworthiness of the kinematic functions and material parameters respectively, and *P*_2_(0) is the kinematics-material correlation sub-matrix, which is a zero matrix in this paper.

### Summary of the algorithmic flow

**Figure 1:**
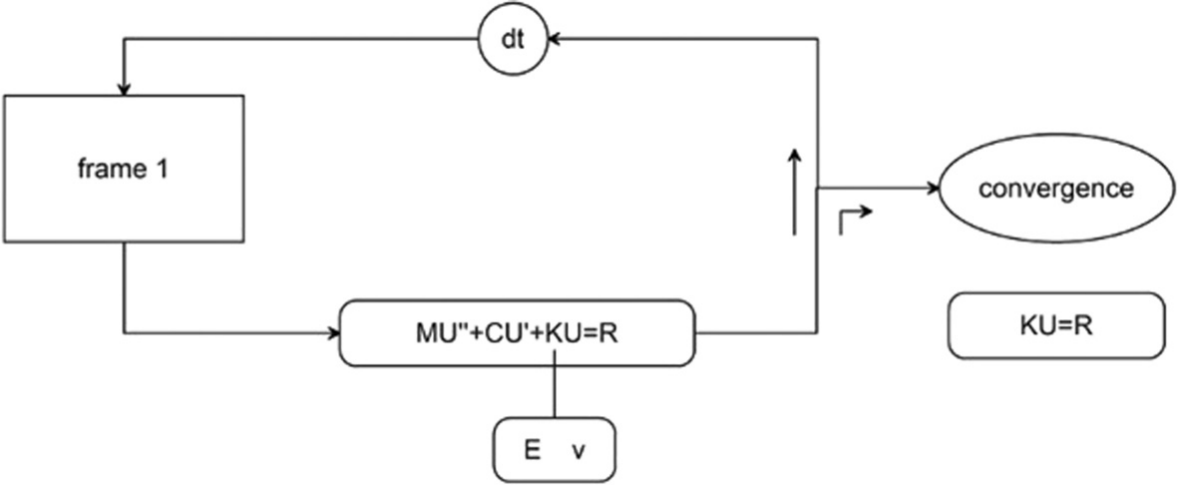
Algorithmic flow chart.

As described above, the system vector *z*, which consists of kinematic vector *x* and material vector *θ*, is updated through the EKF filtering framework, but in this implementation we only have one set of data from quasi-static elastography, i.e., one frame of displacement measurement acquired in deformed configuration. In Figure 1, the flow chart of our filtering framework is illustrated. If the difference between the estimate of current time step and the estimate of previous time step is small enough, the operation of EKF will be terminated and the final estimate will be considered as close enough to the optimal estimation. Therefore, through our filtering framework, a quantification of the internal material properties distribution jointly with kinematic state could be recovered from the ultrasonic RF data collected from quasi-static elastography.

## Experiments and results

In order to demonstrate the performance of our filtering framework, we design three groups of experiments using synthetic data, phantom data and clinical data respectively. All the codes in the following experiments are implemented in Matlab R2013a (MathWorks Corporation, USA) and run in a desktop computer with a core 2 intel CPU and 12G memory.

### Synthetic data

The tissue examined in the quasi-static ultrasound elastography is a three dimensional object, but the elastographic image collected by the ultrasound probe is two dimensional data. Hence, in the first experiment, the scanned area of elastography is treated as one rectangular area, which consists of two distinguished parts (inclusion and background) with different materials parameters (*E*_*y*_=15KPa and *ν*_*y*_=0.47 for the inclusion part, and *E*_*b*_=7KPa and *ν*_*b*_=0.47 for the background part). In order to generate authentic ground truth, the mechanical deformation of the cylinder is simulated in ABAQUS (DS Simulia Corporation, USA) as the ground truth: a constant pressure *P*=1KPa is then applied on the top of the cylinder, and then the deformation ratio is calculated using the maximized displacement of the top boundary divided by the height of cylinder, which is 3% in this simulation. Then deformation of the rectangular cross-section of cylinder is extracted out from the simulation: the rectangular cross-section of cylinder is first divided into a triangular mesh consisted of 231 nodes and 400 triangular elements; then, the displacements and velocities of 231 nodes are exported from ABAQUS as the ground truth. Because the deformation in this experiment is small, we set right-angled equilateral triangle elements in the ABAQUS to do the FEM analysis. During the experiment of our model-based algorithm using the synthetic data, Young’s modulus is treated as a constant inside each triangular element, but the value of Young’s modulus can vary from element to element. Hence, the values of Young’s modulus of those 400 elements are estimated jointly with kinematic functions through our filtering framework.

**Figure 2:**
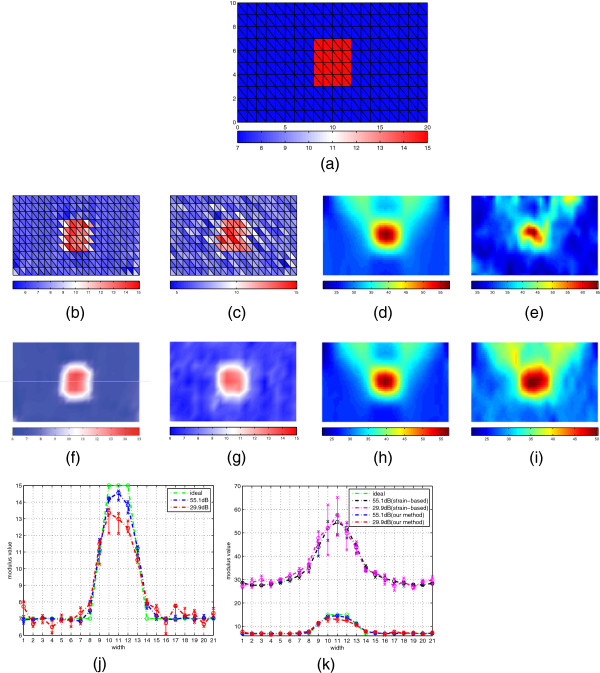
**Experiment on simulated data.** Experimental results in the simulated data: **(a)** ground truth; **(b)** estimated Young’s modulus (55DB); **(c)** estimated Young’s modulus(29.9DB); **(d)** noisy strain map(55DB); **(e)** noisy strain map(29.9DB) ; **(f)** interpolated result(55DB); **(g)** interpolated result (29.9DB); **(h)** estimated strain map(55DB); **(i)** estimated strain map(29.9DB); **(j)** comparison of our method’s performance under different noise conditions; **(k)** performance comparison between our method and strain-based method under different noise conditions.

**Figure 3:**
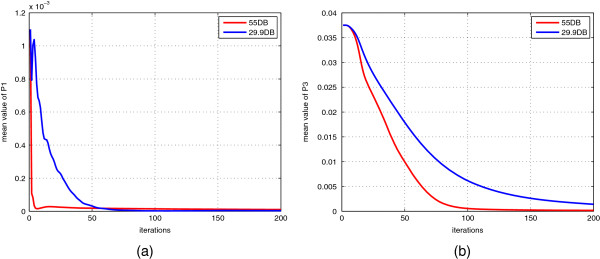
**Converge curve of mean value of *P*1 and *P*3.** The converge curve of mean value of *P*1 and *P*3 in different noise conditions: **(a)** the converge curve of mean value of *P*1 under two different noise conditions; **(b)** the converge curve of mean value of *P*3 under two different noise conditions.

To evaluate the performance of the framework under different noises, two sets of simulated displacement data with different signal-to-noise ratio (SNR) ((1) 55.1*dB*; (2) 29.9*dB*) are generated from the ground truth in this way: 1) simulated displacement data are exported from ABAQUS; 2) two levels of white Gaussian noise are added into the simulated displacement data. It is convenient to generate simulated displacement with different SNR in Matlab platform, such as using wgn function or awgn function. The axial strain maps calculated from noisy displacement data are first inverted as the relative Young’s modulus as the output of a conventional strain-based algorithm with the assumption of stress uniformity [2] as the comparison to the results of the following filtering experiments ((j) and (k) of Figure 2). The estimated results of Young’s modulus over triangular mesh using 55.1*dB* and 29.9*dB* data are shown in (b) and (c) of Figure 2 without smoothness. Interpolated results of Young’s modulus for 55.1*dB* and 29.9*dB* data are illustrated in (f) and (g) of Figure 2 respectively over the triangular mesh. In order to evaluate the effect of different noises on our framework, the estimated strain maps of our filtering framework are illustrated in (h) and (i) of Figure 2, in comparison to the noisy strain maps generated from the ground truth in (d) and (e) of Figure 2. In (j) of Figure 2, the middle line of (f) and (g) of Figure 2 and the deviation of neighboring zone (i.e., the distribution of modulus along the center zone), are illustrated. In (k) of Figure 2, the middle line and the deviation of neighboring zones of our filtering framework and the relative Young’s modulus from the conventional strain-based method are compared together. For two groups of simulated data using in our filtering method, the mean values of *P*1 and *P*3, parts of error covariance matrix *P*, are calculated in every iteration and displayed in Figure 3.

### Phantom data

**Figure 4:**
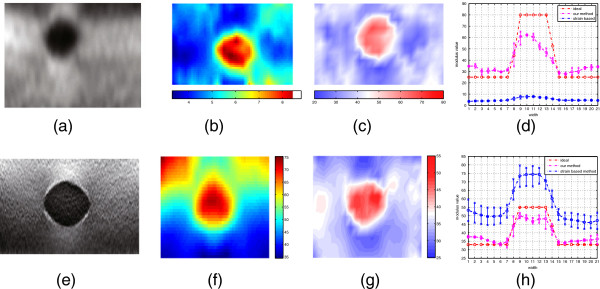
**Experiment on phantom data.** Phantom data: **(a)** strain image of first set of data; **(b)** relative Young’s modulus of first set of data; **(c)** estimated Young’s modulus of first set of data; **(d)** the comparison of Young’s modulus profile along the central line between conventional strain-based method and our filtering framework for first set of data; **(e)** strain image of second set of data; **(f)** relative Young’s modulus of second set of data; **(g)** estimated Young’s modulus of second set of data; **(h)** the comparison of Young’s modulus profile along the central line between conventional strain-based method and our filtering framework for second set of data.

After testing our filtering framework in the simulated data, two sets of real ultrasonic RF data collected from different phantoms are used to evaluate the performance of our filtering framework in this section. The first set of data is collected in one Elasticity QA Phantom (Model 049, CIRS Inc. USA) using a PC-based Ultrasonix RP ultrasound machine (Ultrasonix Medical Corporation, Burnaby, BC, Canada). The true distribution of Young’s modulus of this set of data is: 80kPa for the inclusion and 25kPa for the background part. The axial components of displacement is extracted out from this set of data using a phase-shift method [53]. The second set of data is provided online in public, which is also collected from another Elasticity QA Phantom [54]. The true distribution of Young’s modulus of this set of data is: 55kPa for the inclusion part and 33kPa for the background part. The two-dimensional displacement filed of the second set of data is also provided online in public by [54]. In the following experiment, the relative Young’s modulus is still calculated by inverting axial strain maps based on the assumption of stress uniformity [2]. In Figure 4, the estimated results of our model-based algorithm, the strain image, the image of strain-based method, and the strain profiles along the center line of strain-based method and our method are displayed together for comparison A quantitative comparison of image contrast is also shown in Table 1. The numbers inside the brackets of Table 1 are mean values of Young’s modulus of the inclusion part and background part. In this table, the ideal contrast is compared to the actual contrast in different deformation ratios for two sets of phantom data.

**Table 1:** Quantitive comparison of phantom data

	**Deformation ratio**	**Actual contrast**	**Ideal contrast**
First set of data	0.22%	1.9714(69/35)	3.2(80/25)
Second set of data	6.33%	1.4706(50/34)	1.6667(55/33)

### Clinical data

Three sets of patients’ ultrasonic RF data are provided online in public [54]. These data were collected from patients undergoing open surgical RF thermal ablation for liver cancer who were enrolled between February 6, 2008 and July 28, 2009. The clinical statuses of these patients are described in detail [54]. The ultrasound experiments were performed with the approval of the Health Science Research Ethics Committee of Johns Hopkins University (JHU), and the participants provided written informed consent before beginning the experiment [54]. According to the previous reports on the mechanical property of liver tissue, the tissue with Young’s modulus above 6KPa can be considered as lesion inside liver [55]. All the patients’ data are processed in the same way as previous experiments in phantom data. Since the axial and lateral displacement measurements have been provided in [54], the extraction of displacement is not necessary in this experiment. The initial conditions for all three sets of data are not exactly the same throughout the experiment though the data acquisition is performed on the same kind of organ tissue using the same equipment. For example, noise matrices *Q*_*v*_ and *R*_*e*_ need to be set empirically. According to our experience in the phantom data, the convergence of our filtering framework is not sensitive to the initial values of elements of system error covariance matrix *P* because *P* can be automatically tuned during the EKF procedure as we have shown in Figure 3 in previous experiments.

**Figure 5:**
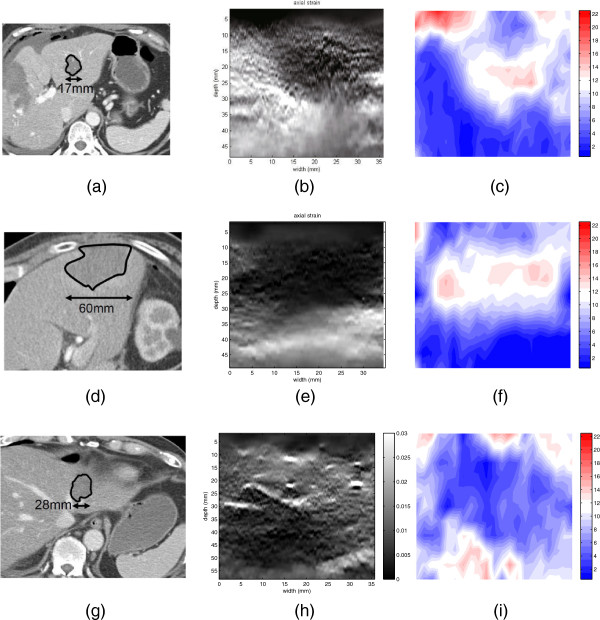
**Experiment on clinical data.** Clinical data: **(a)** CT scan of patient 1; **(b)** strain map of patient 1; **(c)** our result of patient 1; **(d)** CT scan of patient 2; **(e)** strain map of patient 2; **(f)** our result of patient 2; **(g)** CT scan of patient 3; **(h)** strain map of patient 3; **(i)** our result of patient 3. The non-unity aspect ratio in the axes of the strain images and elasticity images should be considered when comparing them to the CT scans, but the axes of the strain images and elasticity images use the unity aspect ration.

In Figure 5, patients’ CT images scanned after RF ablation, strain images and our estimated results are illustrated respectively. In Figure 5, patients’ CT images scanned after RF ablation and strain images are provided online in public by [54]. In CT images, the details of the organ structure is visible, but it is difficult to tell the abnormal tissue from the normal tissue without clinical experiences. The position of tumors in CT images are marked by clinical experts after RF ablation. However, in the strain images provided by [54], it seems that the zones of abnormal tissue are much larger than the markers in CT images. However, the zones of abnormal tissue in our results are clearly smaller than those in the images generated by the strain-based method.

## Discussion

In this paper, a filtering framework is proposed to recover the distribution of Young’s modulus (i.e., a meaningful elastographic image), from sparse and noisy ultrasonic RF data using a biomechanical constraint. The experiments reported in this paper reveal several interesting aspects of our filtering framework, and potentially useful characteristics to solve the recovery problem of elasticity distribution in quasi-static ultrasound elastography. In general, the quality of estimated results is mainly influenced by following factors: noise, deformation ratio, boundary conditions, input information and computational cost.

Two kinds of noises have been studied above: measurement noise and process noise. In most of previous iterative approaches, the measurement noise is considered, but the process noise is always ignored. The main contribution of this paper is to treat the measurement noise and process noise as Gaussian noises. The process noise is caused by the mismatch between the data and the system model, i.e., model-data discrepancy. Since there are not much previous works to study the properties of process noise, the process noise is modeled as white noise distribution, which is the contribution of this paper for iterative approaches to quasi-static elastography to our knowledge. The measurement noise is also modeled as another independent white noise. Therefore, the tolerance of noises of our model-based algorithm is one important factor that should be carefully examined. In the experiment of simulated data, as you can see in (j) of Figure 2, the tolerance of noise of our filtering framework gets worse when the noise becomes larger. Although our filtering method has incorporated the biomechanical constraints, the quality of estimation is still compromised by the noise as shown in (j) of Figure 2, which can be explained by the shortcoming of EKF: when the system is nonlinear, EKF might only be able to give sub-optimal estimates, as it relies on linearization of the nonlinear system equation to propagate the mean and covariance of the state. But when the results of our model-based algorithm are compared to the results of conventional strain-based method, as you can see in (k) of Figure 2, the relative elastogram (i.e., relative Young’s modulus calculated from the conventional strain-based method), is far away from the ground truth and easily corrupted by the noise, while our model-based method still can recover a meaningful elastogram by introducing a biomechanical model into EKF framework. In Figure 4, the results of strain-based method and our filtering framework for the phantom data are displayed and compared. As shown in (d) and (h) of Figure 4, our filtering framework still can generate meaningful distribution of Young’s modulus, but the conventional strain-based method failed to recover the true value of Young’s modulus from ultrasonic data. A quantitative comparison is also shown in Table 1: the values calculated by the ground truth are close to the values calculated by our estimated results. Hence, we can conclude that our filtering method can generate more meaningful results under the assumption of Gaussian noise, which is to say that our filtering method is more robust than the conventional strain-based method in handling the noise from ultrasound elastography.

One factor to compromise the quality of the results of our model-based algorithm is the measurement noise. Measurement noise is produced during the process of acquiring RF data from organ tissue. It is therefore necessary to take the measurement noise effect into account during the recovery procedure. In the past, some of Direct approaches ignore the measurement noise, and their results are drastically affected. The measurement noise introduced during the acquiring process of ultrasonic RF data mainly depends on ultrasound machine and the skill level of operators. One way to increase the SNR is to employ a large deformation ratio during the elastography. That is revealed in phantom study: with larger deformation ratio, the result of the second set of data is better. As shown in the Table 1, the quality of estimation is comprised by the noise largely under a small deformation ratio, therefore a large deformation ratio can be helpful for a quick and quantitive identification of abnormal tissue as shown in Table 1. However, it is not always practical to increase the deformation ratio arbitrarily because of de-correlation of RF signal, so the reduction of the noise effect is still an important issue that still needs to be addressed. Moreover, as shown in (d) and (h) of Figure 4, and Table 1, though our filtering framework is still able to recover a reasonable distribution of Young’s modulus from real ultrasonic data, the quality of estimated results is not so good as the results in the simulated data because the statistical property of system and measurement noises are available in controlled simulation and not easily available during the actual process of elastography. Therefore, the statistical property of process noise (i.e., model-data discrepancy), and measurement noise are worthy to be carefully examined in the future work.

The way to enforce the boundary conditions is also important in our filtering framework. In this work, we have employed three different strategies to enforce the boundary conditions: known surface force distribution (simulated data), penalty method [44] and modifying stiffness matrix *K* with boundary nodes’ displacement information [44]. With known surface force distribution, it is convenient to enforce this condition through external loading *R*. However, surface force distribution introduced by the probe is difficultly acquired in clinical cases. In the penalty method, a large constant, the penalty factor, is added to the diagonal elements of stiffness matrix K, but it will change the structure of the state-space equation and easily diverge our model-based algorithm. One possible way is to use the movement of boundaries of the interested area as the boundary conditions, so the external force conditions can be implicitly enforced by enforcing the movement of boundaries of the interested area. In the phantom and clinical data study, the stiffness matrix *K* is modified by the boundary nodes’ displacement information, which is an alternative way to enforce the boundary conditions without changing the structure of the state-space equation. In our experiments of phantom and clinical data, promising results have proved the efficiency and accuracy of modifying stiffness matrix *K* with boundary nodes’ displacement information. However, the displacement of moving boundary is extracted out from noisy ultrasonic data. So the quality of estimated results of our filtering framework can be affected by the noise of moving boundary from ultrasonic data.

Our filtering method is to generate the optimal estimates using the biomechanical model constraint and the available measurements. In the experiment of phantom data, as explained above, a full displacement field is provided in the second set of data, but only the axial displacement is given in the first set of data. Since the measurement from the second set of data can provide more reliable observations, our filtering framework can generate a better result in the second set of data than that in the first set of data as shown in Figure 4. Hence, in order to maximize the performance of our filtering framework, more reliable observations (i.e., input information to our filtering framework), should be secured. We should point out that the strain and elasticity images without anatomical information will prevent people from upstanding its benefits in clinical practice. Hence, we believe that a further study of the registration of anatomical images (such as CT) and functional images (strain and elasticity images) should be carried on. We admit that it is so difficult to obtain the ground truth in the clinical experiment, and thus it will be always a challenging and interesting topic to carry further study of the performance of our framework using different clinical data.

The initial conditions for all three sets of data are not exactly the same throughout the experiment though the data acquisition is performed on the same kind of organ tissue using the same equipment. For example, noise matrices *Q*_*v*_ and *R*_*e*_ need to be set empirically. According to our experience in the phantom data, the convergence of our filtering framework is not sensitive to the initial values of elements of system error covariance matrix *P* because *P* can be automatically tuned during the EKF procedure as we have shown in Figure 3 in previous experiments. For system error covariance matrix *P*, two important parts, *P*1 and *P*3, should decrease when our filtering method converges. As shown in Figure 3, the mean value of *P*1 and *P*3 decreases through iterations as expected, which indicates that the stochastic dynamic vector and material parametric vector becomes close to the optimal estimates. However, the convergence rate of mean value of *P*1 and *P*3 in small noise condition is higher than under higher noise condition, which is to say that the final converged state is still affected by the noise.

The computational cost is always a problem to the iterative approaches. In our filtering framework, the multiplication of large matrices is a time-consuming task during the iterative process of EKF. In our study, the mesh size is 21*11. The size of state vector is 1324*1, and the size of error covariance matrix is 1324*1324. One loop (Eqs. (22)–(26)) costs about 5-8 seconds, and 200 or more loops are required to reach the convergence of EKF as show in previous Figure 3. It totally takes 15-25 minutes to converge, which is not suitable for real-time data acquisition. Therefore, it is not feasible to use fine mesh in our framework in current hardware configuration. However, after incorporating the biomechanical model as the constraint into our filtering framework, it is clear in Figure 5 that the elastogram map with low sampling rate is much more readily identifiable than the strain map with high sampling rate: the size of lesions are much smaller than that indicated from strain-based images. So considering the quality of our filtering framework for the quasi-static ultrasound elastography, the reduction of computation, such as GPU acceleration of matrix operation, will be a promising researching topic in the future.

## Conclusions

In our filtering framework, the absolute values of Young’s modulus are estimated through the EFK in a MMSE sense. We address the model-data discrepancy and measurement noise by introducing process noise and measurement noise in our framework. Three groups of data: synthetic data, phantom data and patient data, are used to validate the performance of our framework. It is straightforward to apply this framework at other occasions of transformation from noisy dynamic information to material parameter distribution, not limited to mere quasi-static ultrasound elastography.

## Abbreviations

MMSE: Minimum mean square error; EKF: Extended Kalman filter; RF: Radio frequency; SFEM: Stochastic finite element method; FEM: Finite element method; FE: Finite element; SNR: Signal-to-noise ratio; JHU: Johns Hopkins University.

## Competing interests

The authors declare that they have no competing interests.

## Authors’ contributions

ML and HZ contributed equally to this work and the manuscript writing as they both conceived of the study, designed the whole work, and drafted the manuscript. JW carried out the experiments on phantom and synthetic data. JY helped to extract the displacement information from clinical data. ZH participated in the design of this study and coordination and helped to draft the manuscript. HL helped to draft the manuscript. All authors read and approved the final manuscript.

## Authors’ information

Minhua Lu and Heye Zhang contributed equally to this work and share first authorship.
